# Depression management and antiretroviral treatment outcome among people living with HIV in Northwest and East regions of Cameroon

**DOI:** 10.1186/s12879-022-07711-w

**Published:** 2022-09-13

**Authors:** Jackson Jr Nforbewing Ndenkeh, Akindeh Mbuh Nji, Habakkuk Azinyui Yumo, Camilla Rothe, Arne Kroidl

**Affiliations:** 1 Research for Development International (R4DI) Foundation, Yaoundé, Cameroon; 2 Center for International Health, University Hospital, Ludwig Maximilian University of Munich, Munich, Germany; 3 Department of Biochemistry, Faculty of Sciences, University of Yaoundé I, Yaoundé, Cameroon; 4 Division of Infectious Diseases and Tropical Medicine, University Hospital, Ludwig Maximilian University of Munich, Munich, Germany; 5 German Center for Infection Research (DZIF), Partner Site Munich, Munich, Germany

**Keywords:** HIV treatment outcomes, Psychoeducation, Interpersonal psychotherapy, Depression, Adherence, Cameroon

## Abstract

**Background:**

Several interventions have shown benefits in improving mental health problems such as depression which is common in people living with HIV. However, there is a paucity of evidence on the effect of these interventions in improving HIV treatment outcomes. This study aimed at bridging this evidence gap and guiding the integration of depression and HIV management, particularly in rural health settings of Cameroon.

**Materials and methods:**

We carried out a cluster-randomized intervention study targeting persons aged 13 years and above who had been on antiretroviral treatment for 6–9 months. Participants were followed up for 12 months during which those in the intervention group underwent routine screening and management of depression. Comparisons were done using the two-way ANOVA and Chi-squared test with significance set at 5%.

**Results:**

Overall, 370 participants with a median age of 39 years (IQR: 30–49) were enrolled in this study. Of these, 42 (11.3%) were screened with moderate to severe depressive symptoms and 41 (11.1%) had poor treatment adherence. There was a significant drop in depression scores in the intervention group from 3.88 (± 3.76) to 2.29 (± 2.39) versus 4.35 (± 4.64) to 3.39 (± 3.0) in controls (p < 0.001) which was accompanied by a drop in the prevalence of moderate to severe depressive symptoms in the intervention group from 9% to 0.8% (p = 0.046). Decreased depression scores were correlated with better adherence scores with correlation coefficients of − 0.191, − 0.555, and − 0.513 at baseline, 6 months, and 12 months of follow-up respectively (p < 0.001) but there was no significant difference in adherence levels (p = 0.255) and viral suppression rates (p = 0.811) between groups.

**Conclusion:**

The results of this study suggest that considering routine screening and management of depression as an integral component of HIV care could positively impact HIV treatment outcomes. However, there is a need for more research to identify the best combinations of context-specific and cost-effective strategies that can impactfully be integrated with HIV management.

*Trial registration* Trial registration Number: DRKS00027440. Name of Registry: German Clinical Trials Register. Date registration: December 10, 2021 (‘retrospectively registered’). Date of enrolment of the first participant: 05/08/2019

**Supplementary Information:**

The online version contains supplementary material available at 10.1186/s12879-022-07711-w.

## Background

Since the advent of antiretroviral therapy (ART), the fight against HIV/AIDS has significantly evolved in Sub-Saharan Africa (SSA) with improvements in clinical outcomes, survival and reduction of transmission [1]. The scale up of ART has substantially improved access to treatment across SSA, increasing ART coverage from 24% in 2010 to 69% in 2019, and resulting in a 37% reduction in AIDS-related deaths [2]. Notwithstanding, the HIV epidemic is far from ending, with SSA still most affected by the pandemic accounting for 67% of cases worldwide [2]. According to the Joint United Nations Programme on HIV/AIDS (UNAIDS), 970,000 new HIV infections were recorded in SSA in 2019 (representing 57% of all new infections worldwide) with West and Central Africa (WCA) accounting for one quarter of these new infections [2]. Cameroon, despite experiencing a drop in HIV prevalence (from 4.1% in 2010 to 3.0% in 2019), still remains one of the countries in WCA with a high burden of HIV with 15,000 new infections and 14,000 AIDS-related deaths recorded in 2019 [3]. Furthermore, of the 500,000 people estimated to be living with HIV in Cameroon, 78% (390,000) know their HIV status, 74% (370,000) are on ART [3], and recent studies have demonstrated viral suppression rates to vary from 72 to 95% [4–8].

Mental disorders occur at a high rate among people living with HIV (PLHIV) and depression is one of the most common mental health disorders diagnosed in this population group [9]. Depression is often associated with poverty, stress related to HIV diagnosis, and HIV-related discrimination and stigma amongst many others [9]. Depression has been further shown in studies from SSA to be independently associated with poor antiretroviral treatment adherence [10–12]. Meanwhile, optimal treatment adherence is needed to achieve viral suppression, reduce disease progression and prevent transmission [13, 14], whereas suboptimal adherence leads to virologic failure, disease progression and potentially acquired and transmitted drug-resistant virus [14]. Previous studies from Cameroon have found depression to be prevalent in a range of 21–63% of PLHIV, which was associated with alcohol abuse, unemployment, younger age, and low CD4 count [15, 16]. Furthermore, a positive effect on self-reported HIV treatment adherence has been reported [17, 18].

Several studies have demonstrated the effectiveness of mental health interventions on mental health outcomes and wellbeing of persons living with HIV, however, there is still a paucity of data on their effect on HIV treatment outcomes [19]. In addition, a majority of these mental health interventions were demonstrated in high-income contexts which represent a considerably small proportion of all persons living with HIV globally [20]. These studies may not translate into the very diverse low- and middle-income context, where factors such as poverty, patterns of the burden of disease, gender norms, discrimination against specific groups, and mental health resources vary greatly [21]. We therefore developed a study focusing on the needs of PLHIV in an African context, and to evaluate context-specific mental health assessments, interventions and their effects on HIV treatment adherence and outcome. We further aimed to integrate depression management in HIV-related healthcare services, particularly in the rural health settings of Cameroon. The aim of this study was to evaluate the effect of depression management on ART adherence and subsequently virological treatment efficacy at 12 months after study inclusion.

## Methods

### Study design and randomization

This was a cluster-randomized, longitudindal intervention study conducted at two district hospitals in Cameroon; Santa District Hospital (SDH) in the North-West Region and Abong Mbang District Hospital (ADH) in the East Region of Cameroon. Both SDH and ADH are health facilities that provide services including comprehensive HIV/AIDS management to semi-urban and mainly rural populations. At ADH, a total of 2462 adult PLHIV (15 years and above) and 79 children (0–14 years) were on antiretroviral treatment, while SDH had a total of 646 adult PLHIV and 24 children on ART at the time of study initiation. Each health facility serves a catchment area of surrounding villages or living areas, which were defined as clusters. Clusters were adopted to prevent contamination bias whereby a participant in the intervention group might influence a participant from the control group because of proximity in their living locations. Before start of study, we randomly assigned clusters either to the intervention or the control arm and were matched with respect to their number of eligible participants prior the start of the study. Overall, 6 clusters were assigned into the intervention, and 6 into the control arm, equally distributed between the ADH and SDH such that participant coming from a particular catchment would automatically belong to one randomization group or the other.

### Study period and population

This study was conducted from August 2019 to July 2021, with recruitment done up to October 2020 and a follow-up done up to July 2021. Individuals were included in this study if they were; (1) aged 13 years or above, (2) HIV positive and had started ART 6–9 months ago at the health facilities, (3) aware of their HIV status and had the autonomy to be actively implicated in their treatment, and (4) they would understand the study intervention. Adults (≥ 21 years) were enrolled upon written informed consent while adolescents (13–20 years) were only enrolled upon informed consent from parent/guardian and assent from the adolescents themselves who in this case ought to be aware of their health situation. For illiterate participants, the study was explained in simple language (which was French, English, pidgin or the mother tongue where possible). Upon acceptance to participate, they were asked to signal their accord by placing some sort of signature or symbol. A participant was considered to have finished study if that participant was followed in the study up to month-12 after enrolment.

### Sample size

The sample size was calculated based on the primary study endpoint which is difference in proportions of participants with unsuppressed viral loads between study arms. We used a general sample size calculated from the formula for comparing two observed proportions which in this case were the viral failure rates.

$${\mathbf{N}}\, = \,[{{\mathbf{P}}_{\mathbf{e}}}({\mathbf{1}} - {{\mathbf{P}}_{\mathbf{e}}})\, + \,{{\mathbf{P}}_{\mathbf{c}}}({\mathbf{1}} - {{\mathbf{P}}_{\mathbf{c}}})/{{{\varvec{\updelta}}}^{\mathbf{2}}}] \times {\mathbf{f}}({{\varvec{\upalpha}}},{{\varvec{\upbeta}}}),$$

where **P**_**c**_ = 17.5%, **P**_**e**_ = 8.75%, **δ** = 8.75% and **f**(**α**, **β**) = 7.9 which gave an estimated total sample size of 232. To ensure that a minimum of 232 participants will reach endpoint of study in spite of lost to follow-up, 50% surplus was added such that a minimum of 350 participants were enrolled in 1:1 allocation between randomization groups.

### Recruitment and data collection at baseline

PLHIV were recruited at their next routine antiretroviral drug refill appointment from start of study. Study information and informed consent procedures were performed by trained research officer and eligible patitents were subesequnetly enrolled and randomized according to their respective cluster assignment. At baseline, we collected sociodemographic information (age, sex, marital status, education level, time to reach treatment center), medical or treatment related information (treatment start date, WHO stage, ART regimen, CD4 counts and routine viral load level if available), and participants’ socio-environmental information (being part of a support group, self-reported relationship with caregivers; psychosocial agents who through task shifting are implicated in HIV management, HIV status disclosure, self-reported level of knowledge of their HIV health status).

### Intervention

All study participants received the standard-of care (SoC) services according to national HIV guideline [22], that included monthly pick-ups for ART, 6-monthly clinical consultations with medical doctors, including health assessments, psychosocial support focused on ART adherence counseling and laboratory workups (safety lab, CD4 counts, viral load). Patients who interrupted treatment received enhanced counselling that focused on identifying the cause while searching for solutions to restart treatment. In addition to SoC, all participants received study specific depression screening at baseline, months 6 and 12 of follow-up. In contrast to the control arm, participants assigned to the intervention arm further underwent quarterly depression screenings. We screened for symptoms of depression in the participants using the standard Patient Health Questionnaire (PHQ-9) which was first validated by Kreonke et al. in the USA [23], then later in South Africa [24] and has since been widely used in SSA. This PHQ-9 constitutes of a series of nine questions seeking to identify the severity of depression symptoms within a period of 2 weeks prior to the visits. The severity of each depression symptom was graded on a rating score 0–3 where a score of two and above meant the symptom was present. The overall rating score was 0–27 based on the following depression symptoms: little or no interest, hopelessness, trouble sleeping or oversleeping, feeling tired, low appetite or overeating, low self esteem, trouble concentrating, slow reaction or restlessness and suicidal thoughts. Depression levels were classified as follows: 0–4 was no symptom of depression, 5–9 was mild symptoms of depression, 10–14 was moderate symptoms of depression, 15–19 was moderately severe symptoms of depression and ≥ 20 was severe symptoms of depression. A participant was thus considered to have moderate to severe depressive symptoms if s/he had a depression score of 10 and above.

In the situation where depressive symptoms were screened in a participant of the control arm, that participant was referred to their healthcare provider (psycho-social agent) who would normally handle such cases within the framework of their psychosocial support. Participants from the intervention arm received psycho-education, and interpersonal psychotherapy (IPT). For severe cases pharmacotherapy was applied. Psycho-education is an intervention with systematic, structured, and didactic knowledge transfer for an illness and its treatment, integrating emotional and motivational aspects to enable patients to cope with the illness and to improve its treatment adherence and efficacy [25]. In this study, it was conducted for participants of the intervention arm with mild symptoms of depression [5–9] by a trained research officer where participants were given key messages on the symptoms of depression, perceptions and misconceptions of the participant as well as their family and friends with regards to depression, when it was important to seek for help, available treatments and their benefits [26]. We further provided information on the key problem areas that can cause depression as well as on HIV management and the association of depression with their care. For participants of the intervention arm with moderate (10–14), and moderately severe (15–19) symptoms of depression, psycho-education was coupled with IPT. The IPT is an intervention conceptualized such that strategies and techniques occur in three phases during which there is diagnostic evaluation, framework for treatment, strategy adaptation with respect to main problem area(s) of depression and consolidating therapeutic gains from those strategies [27]. This IPT was conducted by the research officer (serving here as therapist) in accordance to the “Comprehensive Guide to Interpersonal Psychotherapy” by Weissman, Markowitz, and Klerman published in 2000 [27]. This guide is simplified such that it can be implemented by non-specialists. However, we used the above guide and further the WHO’s Mental Health Gap Action Programme (mhGAP) intervention guide [26] to make a summarized standard operating procedures (SOP) used by the research officers. The very first visit served as an opportunity to perform psycho-education which was the prelude to IPT. This first visit also served to identify the main problem area(s) faced by the participant (maximum of two from role transition, grief, interpersonal disputes and interpersonal deficits). During subsequent visits, in addition to continuous education and psychosocial support, the therapist would emphasize to the participant that depression is due to present problems they are currently facing and therefore solutions can be found which would help participants to avoid blaming themselves for any of the above identified problem area(s). The therapist together with the participant identified possible social withdrawals, losses, and altered relationships in the participant's life, also focusing on possible solutions as applicable. The participant was helped to deal with their depressive state by adopting problem area specific stretegies, for instance role transition, recognizing the positive and negative aspects of the new role, as well as the assets and liabilities of the old role this one replaces. Furthermore, these patients were contacted by the therapist (direct calls or calls through another close family member or friend aware of the patient’s situation) for follow-up in case the participant was unable to attend IPT session appointments. To ensure that there was no bias in the knowledge acquired and to the service package delivered, both research officers were trained at the same time, provided with the same version of the SOP above as well as had frequent virtual and in-person feedback sessions with the principal investigator where they discussed on the various IPT sessions they had conducted. For any participant of the intervention arm screened with severe symptoms of depression (≥ 20), both psycho-education and IPT were used and as needed coupled with pharmacotherapy, whereby a medical doctor at the health facility would prescribe Amitriptyline to the participant.

Participants of the intervention group with depressive symptoms at baseline, received the intervention throughout the 12 months follow-up period. Participants with depressive symptoms within the routine follow-up screening, received the intervention throughout the remainder follow-up period. The intervention was thus conducted and re-adapted each time with respect to change in follow-up depression score until symptoms resolved (i.e., depression score of 0–4). Participants of the intervention arm thus had a minimum of five standard visits during which depression assessments and management were done. The frequency of IPT sessions was guided by participant’s drug refill appointments and/or general availability in an attempt to align the therapy sessions with the participant’s routine. Each IPT session lasted for a minimum of 30 min.

### Outcome measures

This study measured the effect of the above intervention on ART adherence levels and on viral suppression. Viral suppression which is the endpoint outcome of HIV treatment was the primary outcome of this study meanwhile treatment adherence which is an intermediate outcome of HIV treatment was secondary outcome. The adherence levels of the participants were assessed using a multi-method adherence measurement tool which had been validated in South Africa records [28]. This tool includes a pill identification test (PIT), 4-items self-reporting adherence questionaire, visual analogue scale (VAS) of adherence within the past 5 days and pill counts from the last drug refill appointment (Additional file 1). In Cameroon, a study by Atanga et al. [29] first used the above multi-method adherence measurement tool, however replacing PIT by drug refill appointments as extracted from pharmacy records [28, 29]. We graded all four components of the above validated multi-method adherence measurement tool and included the pharmacy records component as shown in Table 1.

**Table 1:** Treatment adherence assessment guide

Individual and overall adherence grading (score)			
**Drug refill**	Had all 6 refills (3)	Missed 1 refill (2)	Missed 2 or more refills (1)
**Pill identification test**	Knows drug, dose and time (3)	Dose and time only (2)	Dose or time only or confused (1)
**Self-reported**	No to all 4 questions (3)	Yes to 1 question (2)	Yes to 2 or more questions (1)
**Visual Analogue Scale**	95% and above (3)	75–94% (2)	Less than 75% (1)
**Pill count**	95% and above (3)	75–94% (2)	Less than 75% (1)
**Overall adherence**	Highly adherent (15)	Moderately adherent (10–14)	Poorly adherent (5–9)

Each of the five adherence tools were summarized providing an overall adherence rating from 5 to 15 with a score of 15 indicating high adherence, 10–14 moderate adherence, and 5–9 poor adherence. For the determinants of poor adherence in our analysis, we focused on the poorly adherent category as compared to the other two adherence categories.

Viral load (VL) samples were collected from all participants at the final 12-months visit, and were transported to the closest reference laboratory, which were the Bamenda Regional Hospital for samples from SDH (GeneXpert® HIV-1 Viral Load, Cepheid) and the Bertoua Regional Hospital for samples from ADH (Generic HIV-1 Charge Virale, Biocentric). As of January 2019, VL testing was made free by the Mininstry of Public Health in Cameroon. VL-values below 1000 copies per ml were considered as suppressed. It is worth noting however that by end of study, not all participants had received their VL results.

### Data management and analysis

Data analysis was done using IBM SPSS Statistics version 23.0 for Windows (IBM Corporation, Armonk, NY, USA) and GraphPad Prism version 9.2.0 for Windows (GraphPad Software, San Diego, California USA). Baseline characteristics of participants were presented as proportions in cross-tabulation with respect to randomization group and were compared using the chi-squared test. Spearman’s rank correlation was used to determine correlations between depression scores, treatment adherence scores and viral load levels. We further used logistic regression to identify potential covariates of poor treatment adherence worth noting. All of these covariates were then adjusted for each other’s effect in a multivariate model. The significance was set at 5%. To account for differences between study sites, the effect of drug type and side effects influencing the observed association between depression and adherence, the above model was also adjusted for Efavirenz-based regimen at start (compared to Nevirapine and Dolutegravir-based regimens), reported undesired drug effect and study site. The depression and adherence scores were presented as means and were compared with respect to randomization group and/or time period of study using the two-way ANOVA test. It is worth noting that depression and adherence scores were compared for all participants in an intention to treat context where all participants’ data were used irrespective of their eligibility for intervention. We then compared only for participants with depressive symptoms who were thus eligible for intervention. The above outcome variables were then regrouped to categorical variables and were compared with respect to randomization group and time period of study using the Chi-square test. The proportion of partipants with unsuppressed viral loads (≥ 1000 copies/mL) were also compared between randomization groups for all participants and only for participants with depressive symptoms using Chi-square test. The significance of the statistical tests was set at 5%.

## Results

### Baseline sociodemographic and medical characteristics

**Fig. 1:**
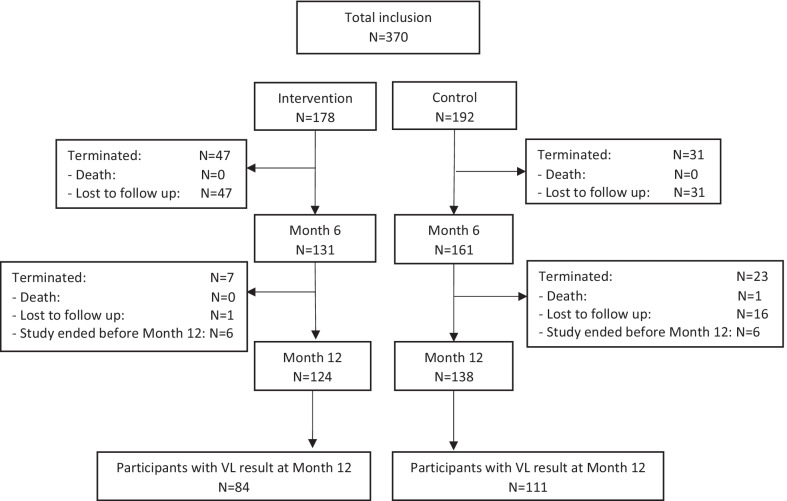
Enrolment and follow up flowchart of study participants

A total of 370 participants were enrolled in the study (59 from SDH and 311 from ADH), of which 178 were assigned to the interevention arm and 192 to the control arm. Overall, 262 participants completed the study by month 12, and 195 with available viral load results by the time study ended, were included into the analysis (Fig. 1). Participants had a median age of 39 years (IQR: 30–49 years), 65.9% were female, and about half (50.7%) were married or in a union (Table 2). Overall, 48% took 30 min to 2 h, and 10% more than 2 h to reach the treatment center, and this was not equally distributed between arms as partcipants assigned to the control arm had greater distances to treatment centers. Concerning the HIV status, more than three quarter of the participants were at WHO disease stages I/II when starting treatment, and the majority (86.1%) started on an Efavirenz based regimen. Also, 35.1% of the participants considered themselves to be quite knowledgeable of their health situation, of HIV pathology, HIV treatment and its effect on their health as well as on appropriate health-behaviors to adopt. Furthermore, 52.2% of participants felt that their relationship to their healthcare provider at the treatment center was more than just medical, implying that visits went beyond their medical and treatment related needs, but also included interest on participant’s social and psychological wellbeing.

**Table 2:** Characteristics of participants at enrolment (N = 370)

Variable	Total^a^	Randomization group Intervention, N = 178; control, N = 192		p
	N (column %)	n (row %)	n (row %)	
**Age in years; median (IQR)**	39 (30–49)	38 (29–48)	40 (32–49)	0.057
**Age group**	370	178	192	0.725
Adolescent (< 20 yrs)	10 (2.7)	6 (60.0)	4 (40.0)	
Adult (20–50 yrs)	289 (78.1)	139 (48.1)	150 (51.9)	
Elderly adult (> 50 yrs)	71 (19.2)	33 (46.5)	38 (53.5)	
**Sex**	370	178	192	0.601
Female	244 (65.9)	115 (47.1)	129 (52.9)	
Male	126 (34.1)	63 (50.0)	63 (50.0)	
**Marital status**	369	178	191	0.122
Single	135 (36.6)	76 (56.3)	59 (43.7)	
Couple	187 (50.7)	83 (44.4)	104 (55.6)	
Divorced/separated	13 (3.5)	5 (38.5)	8 (61.5)	
Widow(er)	34 (9.2)	14 (41.2)	20 (58.8)	
**Time to reach treatment center**	369	178	191	< 0.001
Less than 30 min	155 (42.0)	131 (84.5)	24 (15.5)	
30 min to 2 h	177 (48.0)	36 (20.3)	141 (79.7)	
More than 2 h	37 (10.0)	11 (29.7)	26 (70.3)	
**HIV status disclosure**	369	177	192	0.588
No, to none of them	85 (23.0)	42 (49.4)	43 (50.6)	
Yes, to some of them	196 (53.1)	97 (49.5)	99 (50.5)	
Yes, to all of them	88 (23.9)	38 (43.2)	50 (56.8)	
**WHO disease stage**	359	174	185	0.299
Stage I and II	318 (88.6)	151 (47.5)	167 (52.5)	
Stage III and IV	41 (11.4)	23 (56.1)	18 (43.9)	
**Drug at the start**	352	169	183	0.161
Efavirenz-based regimen	303 (86.1)	144 (47.5)	159 (52.5)	
Nevirapine-based regimen	25 (7.1)	16 (64.0)	9 (36.0)	
Dolutegravir-based regimen	24 (6.8)	9 (37.5)	15 (62.5)	
**Level of knowledge on health status**	367	177	190	0.206
Basic knowledge on HIV and treatment	238 (64.9)	109 (45.8)	129 (54.2)	
Extensive knowledge on HIV and treatment	129 (35.1)	68 (52.7)	61 (47.3)	
**Qualify relationship with caregiver**	370	178	192	0.301
More than medical	193 (52.2)	98 (50.8)	95 (49.2)	
Not only medical but not quite good	48 (13.0)	25 (52.1)	23 (47.9)	
Strictly medical	129 (34.9)	55 (42.6)	74 (57.4)	

p is Chi-squared p-value for differences between randomization groups or Mann Whitney test

^a^Not all totals of observations sum to 370, this is due to missing data at that level for some participants

### Depressive symptoms at baseline and evolution over time

**Fig. 2:**
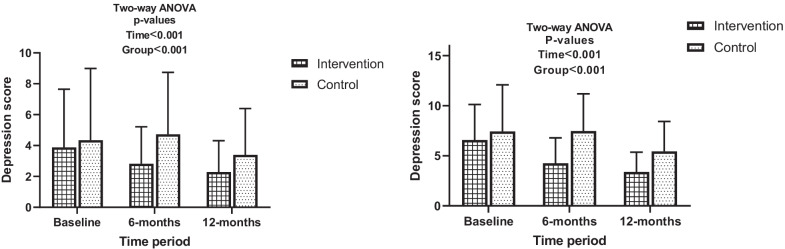
Effect of intervention on depression over time (left: all study participants, right: participants with depressive symptoms in study)

At baseline, 42 (11.4%) of the participants were screened with moderate to severe depressive symptoms overall, among them 31 (8.4%), 10 (2.7%) and 1 (0.3%) presented with moderate, moderately severe and severe symptoms of depression respectively. Between the intervention and control groups, no significant difference was observed with respect to depression categories at baseline (Table 3). However, depression scores changed significantly between groups over the follow-up periods of the study (Fig. 2). For all participants of the intervention arm, the mean depression scores steadily decreased from 3.88 (± 3.76) at baseline to 2.82 (± 2.39) at 6 months of follow-up then to 2.29 (± 2.39) at 12 months of follow-up. All participants of the control arm had mean depression scores which increased from 4.35 (± 4.64) at baseline to 4.73 (± 4.01) at 6 months follow-up then dropped to 3.39 (± 3.0) at 12 months of follow-up. When focusing only on participants with depressive symptoms, same trends were observed. Participants who received intervention had a drop in their depression scores from 6.58 (± 3.55), to 4.26 (± 2.25) then to 3.39 (± 1.98) while controls had 7.43 (± 4.66), 7.48 (± 3.71) and 5.44 (± 2.99) at baseline, 6 months and 12 months of follow-up, respectively. These observed changes in mean depression scores were statistically significant with respect to randomization group and follow-up periods, irrespective of analysis on all participants or just on those with depressive symptoms. When compared in clinical categories, the prevalence of moderate to severe depressive symptoms significantly dropped from 16 (9%) at baseline to 2 (1.6%) at 6 months follow-up and then to 1 (0.8%) at 12 months follow-up in the intervention arm as opposed to 26 (13.5%), 27 (16.8%) and 7 (5.1%) at baseline, 6 months and 12 months of follow-up respectively in the control arm (Table 3).

**Table 3:** Depression, treatment adherence, and viral load suppression outcomes

Variable	Time period								
	Enrolment (N = 370)			6 months (N = 292)			12 months (N = 262)		
	Intervention N (column %)	Control N (column %)	p	Intervention N (column %)	Control N (column %)	p	Intervention N (column %)	Control N (column %)	p
**Depression status**	178	192	0.271	129	161	< 0.001	122	137	< 0.001
No symptom of depression	106 (59.6)	116 (60.4)		98 (76.0)	93 (57.8)		111 (91.0)	98 (71.5)	
Mild symptoms of depression	56 (31.5)	50 (26.0)		29 (22.5)	41 (25.5)		10 (8.2)	32 (23.4)	
Moderate symptoms of depression	14 (7.9)	17 (8.9)		2 (1.6)	24 (14.9)		1 (0.8)	7 (5.1)	
Moderately severe symptoms of depression	2 (1.1)	8 (4.2)		0 (0)	3 (1.9)		0 (-)	0 (-)	
Severe symptoms of depression	0 (0)	1 (0.5)		0 (–)	0 (–)		0 (–)	0 (–)	
**Has moderate to severe depressive symptoms?**	178	192	0.168	129	161	< 0.001	122	137	0.046
No	162 (91.0)	166 (86.5)		127 (98.4)	134 (83.2)		121 (99.2)	130 (94.9)	
Yes	16 (9.0)	26 (13.5)		2 (1.6)	27 (16.8)		1 (0.8)	7 (5.1)	
**Adherence status**	178	192	0.649	131	161	0.098	124	138	0.255
Poorly adherent	20 (11.2)	21 (10.9)		0 (0)	4 (2.5)		0 (0)	3 (2.2)	
Moderately adherent	114 (64.0)	131 (68.2)		94 (71.8)	122 (75.8)		86 (69.4)	94 (68.1)	
Highly adherent	44 (24.7)	40 (20.8)		37 (28.2)	35 (21.7)		38 (30.6)	41 (29.7)	

p is the Chi-squared p-value for differences in outcomes between strata

### Association between depression and treatment adherence

Depression scores were observed to be signifcantly correlated to adherence scores with Spearman’s correlation coefficients of − 0.191, − 0.555 and − 0.513 at baseline, 6 months follow-up and 12 months follow-up respectively (p < 0.001). Furthermore, both depression and adherence scores were observed to be significantly correlated to viral load levels at 12 months of follow-up with Spearman’s correlation coefficients of 0.191 (p = 0.008) and − 0.232 (p = 0.001) respectively (Table 4). In an adjusted regression analysis (Table 5), participants screened with moderate to severe depressive symptoms were more likely to have poor treatment adherence compared to those without (aOR = 5.5; 95% CI = 1.45–20.93; p = 0.012). Also, participants who felt that their relationship with their caregivers was more than just medical (i.e., the caregiver usually went further to enquire and show interest on participant’s social and psychological wellbeing) were less likely to have poor treatment adherence at baseline (aOR = 0.08; 95% CI = 0.02–0.28; p < 0.001).

**Table 4:** Correlation between depression, adherence and viral load

	Adherence at baseline	Adherence at 6 months	Adherence at 12 months	Viral load at 12 months
**Depression score at baseline**				
r	− 0.191	NA	NA	NA
p-value	< 0.001			
N	368			
**Depression score at 6 months**				
r	NA	− 0.555	NA	NA
p-value		< 0.001		
N		290		
**Depression score at 12 months**				
r	NA	NA	− 0.513	0.191
p-value			< 0.001	0.008
N			259	192
**Viral load at 12 months**				
r	− 0.12	NA	− 0.232	NA
p-value	0.095		0.001	
N	194		195	

*r* Spearman’s correlation coefficient

**Table 5:** The association of various potential risk-factors with poor adherence at baseline (N = 346)

Model covariates	Unadjusted		Adjusted	
	OR (95% CI)	p	aOR (95% CI)	p
**Has moderate to severe depressive symptoms?**^a^		0.086		0.012
Yes	2.1 (0.9–4.92)		5.5 (1.45–20.93)	
No	1		1	
**Age group**		0.027		0.195
Adolescent (< 20 yrs)	7.22 (1.59–32.91)	0.011	4.75 (0.67–33.54)	0.118
Adult (20–50 yrs)	1.3 (0.52–3.25)	0.572	0.96 (0.32–2.92)	0.941
Elderly adult (> 50 yrs)	1		1	
**Sex**		0.29		0.213
Female	0.7 (0.36–1.36)		0.6 (0.27–1.34)	
Male	1		1	
**Level of knowledge on health status**		0.03		0.966
Basic knowledge on HIV and treatment	2.43 (1.09–5.43)		1.02 (0.38–2.77)	
Extensive knowledge on HIV and treatment	1		1	
**HIV status disclosure**		0.078		0.452
No, to none of them	3.79 (1.18–12.14)	0.025	2.42 (0.61–9.58)	0.208
Yes, to some of them	2.93 (0.99–8.72)	0.053	1.95 (0.53–7.13)	0.311
Yes, to all of them	1		1	
**Qualify relationship with caregiver**		< 0.001		< 0.001
More than just medical	0.14 (0.06–0.32)	< 0.001	0.08 (0.02–0.28)	< 0.001
Not only medical but not quite good	0.22 (0.06–0.76)	0.017	0.26 (0.06–1.08)	0.064
Strictly medical	1		1	
**Time to reach treatment center**		0.008		0.108
Less than 30 min	0.31 (0.13–0.76)	0.011	0.38 (0.13–1.11)	0.078
30 min to 2 h	0.25 (0.1–0.62)	0.003	0.33 (0.11–1.01)	0.05
More than 2 h	1		1	

NB: adjusted model includes all variables shown and was additionally adjusted for Efavirenz-based regimen at start, reported undesired drug effect and study site as all three were potential confounding factors of the observed association between depression and poor adherence

^a^Variable of interest adjusted for other variables worth noting and variables related to it in the multivariate model

### The effect of depression management on treatment adherence

**Fig. 3:**
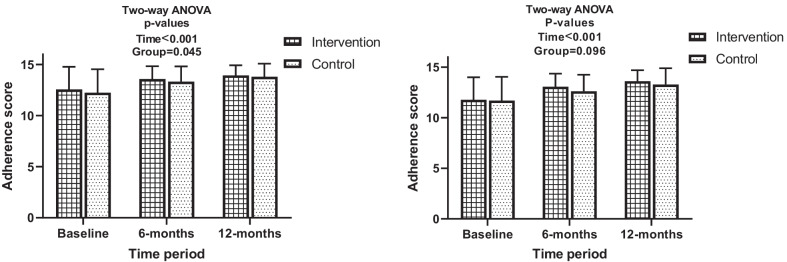
Effect of intervention on treatment adherence over time (left: all study participants, right: participants with depressive symptoms in study)

At baseline, 41 (11.1%) of the participants had poor treatment adherencce levels while 245 (66.2%) and 84 (22.7%) had moderate and high treatment adherence levels respectively. No significant difference was observed between intervention and control groups with respect to adherence levels at baseline (Table 3). Adherence scores changed significantly with respect to randomization group over the follow-up periods of the study (Fig. 3). All participants of intervention and control arms both saw their mean adherence scores increase from 12.56 (± 2.2) and 12.25 (± 2.29) at baseline to 13.58 (± 1.26) and 13.33 (± 1.5) at 6 months of follow-up and to 13.94 (± 0.99) and 13.8 (± 1.28) at 12 months respectively. When focusing only on participants with depressive symptoms, same trends were observed. Participants from the intervention and control arms both had an increase in their mean adherence scores from 11.78 (± 2.23) and 11.7 (± 2.35) at baseline to 13.08 (± 1.29) and 12.62 (± 1.65) at 6 months of follow-up then to 13.62 (± 1.08) and 13.29 (± 1.61) at 12 months of follow-up. Change in treatment adherence levels over time were not statistically significant, irrespective of analysis on all participants or just on those with depressive symptoms. However, all partcipants with poor adherence (N = 4 at 6 months and N = 3 at 12 months) were from the control arm of the study (Table 3).

### The effect of depression management on viral load suppression

**Fig. 4:**
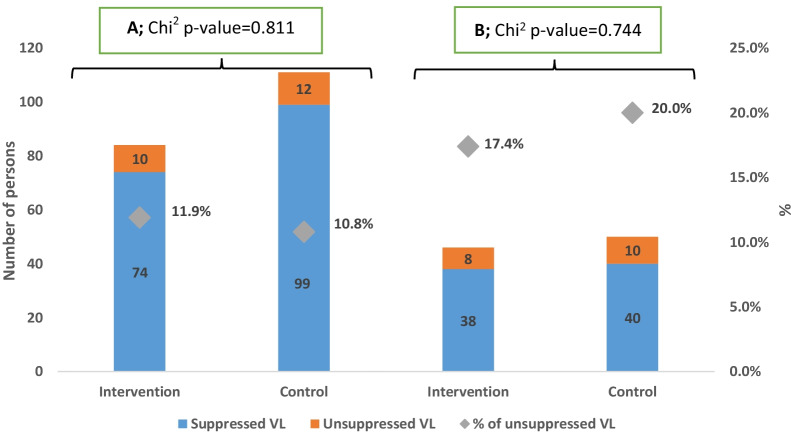
Effect of intervention on viral load suppression at 12 months of follow-up (**A** all study participants, **B** participants with depressive symptoms in study)

By the end of the study, 262 (70.8%) of the participants had reached 12 months of follow-up all of whom had sample collected for viral load test. However, 195 (74.4%) of these participants had received their viral load results. As shown on Fig. 4, the proportion of unsuppressed viral loads (VL ≥ 1000 copies/mL) between participants from the intervention and control arms did not differ between groups, irrespective of analysis on all participants or just on those with depressive symptoms (All study participants; 11.9% versus 10.8%, p = 0.811: participants with depressive symptoms in study; 17.4% versus 20.0%, p = 0.744).

## Discussion

Few studies have reported on the effect of depression management interventions, notably using the interpersonal psychotherapy (IPT), on HIV treatment outcome in Africa, especially in Cameroon. This study thus provides insights on the effect of a pre-defined intervention on depression among PLHIV as well as on their HIV treatment adherence and virological outcome, which can guide its integration in HIV management within the country and subregion.

In our study we observed poor treatment adherence after 6–9 months of ART initiation in 11.1% of all participants while 66.2% and 22.7% were moderately and highly adherent respectively. Our result on poor adherence is similar to that of Atanga et al. (2018) who reported a poor adherence rate of 9.6% at 6 months among pregnant women in Prevention of Mother to Child Transmission (PMTCT). However, this author reported moderate and high adherent levels at 31.4% and 59.0% respectively, which are slightly different from our results [29]. These observed differences could be explained by the study being conducted only in women within the PMTCT (Option B+) program with more deployed resources as well as stringent monitoring. Studies conducted in other SSA countries and that used visual analogue scale and pill counts to determine HIV treatment adherence had fairly similar results to our study. For example, in Southwest Nigeria, Afe et al. [30] using pill counts observed that 8.4% of participants had poor adherence. Another study by Bijker et al. [31] using visual analogue scale in an attempt to compare between Africa and Asia observed a poor adherence rate of 7.3% overall in six African countries. This shows that treatment adherence is fairly good but that there is still need for some adherence enhancing strategies for those with suboptimal adherence levels while giving particular attention to those with poor treatment adherence. The retention rate in our study was 74% after 12 months of-follow-up which is within the range of rates reported from other studies in Cameroon: 78.8% observed after 24 months by Ajeh et al. [32], and 60.4% observed nationwide by Billong et al. [33] after 12 months of care [32, 33].

We further found that 6–9 months after ART initiation, 11.4% of all participants had moderate to severe depressive symptoms and that these participants were about five times more likely to have poor treatment adherence levels as compared to those without (p = 0.012). The prevalence of moderate to severe depressive symptoms in this study was close to the 9% determined by Wagner et al. in Uganda (2017) and the estimated 13–24% in a systematic review in SSA by Bernard et al. in 2017 [12, 34]. The above prevalence of moderate to severe depressive symptoms was however lower than the range of 21–63% from previous studies in Cameroon [15–18, 35–37]. When considering the cutoff score of ≥ 10 as in this study, the prevalence observed by Ngum et al. [37] was even lower with 3.3% of all participants. These differences could be due to background socio-economic and cultural differences of the various study populations as well as the differences in the time of treatment before enrolment in study. It should be noted however that despite having a sensitivity of 88% and a specificity of 88% to detect depression, the PHQ9-questionnaire (score ≥ 10) has limitations in identifying depressed individuals with dysthymia thus depression could be underdiagnosed [38]. The advent of moderate to severe depressive symptoms was independently associated with poor adherence in our study as has been shown by several studies conducted in SSA [10–12, 37, 39, 40]. In this light the World Health Organization in its most recent consolidated guidelines has re-iterated the importance of integrating depression assessment and management in the package of health services for all individuals living with HIV [19], which is supported by acceptability, potential impact on treatment engagement and overall wellbeing of PLHIV [41–43].

Notwithstanding, the paucity of information on the most appropriate depression interventions and their long term impact on depression management and/or HIV treatment outcome still remains [19, 44]. Despite the undisputed effect that anti-depressants (pharmacotherapy) have on the mental health and wellbeing of PLHIV [17], this adds to another long-term treatment that is potentially associated with additional side effects and drug interactions [45]. In this respect behavioral interventions like psycho-education and interpersonal psychotherapy have been proven to be as efficacious as pharmacotherapy and can be used in combination with the above in an attempt to effectively treat depression [45–48]. In this study, we focused on the two behavioral interventions above and only considered pharmacotherapy when a participant of the intervention arm was screened with severe symptoms of depression. Even though the depression management interventions were not received by all participants of the intervention arm nor at the same standard in terms of frequency, we could demonstrate that the intervention had a moderate but significant impact on depression. Moreover, overall improved depression scores were significantly correlated with better adherence over time for all participants. However, we could not detect significant differences in adherence levels between the intervention and control arms despite significant impact of our intervention package. Additionally, we could not find a significant difference between arms on the viral suppression rates after 12 months of follow-up. This could suggest that depression management should not be considered as a stand-alone solution to improve treatment adherence and viral suppression in PLHIV, as other socioeconomic, cultural and medical factors might also have impact. In this respect we also found a significant association between treatment adherence and the self-reported quality of relationships with caregivers. Martin et al. [49] clearly underlines that it is important for the healthcare professional to know the patient as a person thus better understand elements that are crucial to that patient’s adherence. This nurtures trust in their therapeutic relationship and in the long run can be quite beneficial in improving patient’s adherence [49]. Another factor worth taking note was the time participants took to reach the treatment center where participants who lived less than 30 min or 30 min to 2 h from the treatment center had less chances of poorly adhering to treatment. Despite not remaining significant when adjusted for other variables in a model, it is worth noting that this variable differed significantly between the study groups. Some studies have shown that for long term treatments, distances to treatment center especially when coupled with frequent visits can negatively impact adherence to treatment as displacement and transportation costs come in play [50–52]. This difference in time to reach treatment center between the study groups could have impacted the comparable adherence levels measured between both study groups. Depression management thus should be combined with other strategies proven to have the same kind of effect on adherence and/or viral suppression in the same or closely related context notably improved access to services, improving communication and quality of interaction with patients among others in this context.

The drop in the prevalence of moderate to severe depressive symptoms in the control group from baseline to month 12 was striking which entails that the psychosocial follow-up received by participants identified with symptoms of depression and referred to their corresponding caregivers for close follow-up potentially influenced their depressive state. This is in line with Stockton et al. [53] who debated on the lack of pure controls to measure intervention effect where patients are neither briefed on depression during screening nor are denied care when screened. This could have diluted the overall effect of the intervention on treatment adherence and/or viral suppression, however, it underlines the importance of routine depression screening for all PLWHIV. Another factor that could also be attributed to is the known neuro-psychiatric side effects related to Efavirenz treatment, which is a component of the antiretroviral treatment regimen for most patients, and that usually weans off with time. However, it is worth noting that study participants had been on treatment for 6–9 months prior their enrolment in the study thus for those who started with Efavirenz, could have already experienced a wean off in its neuro-psychiatric side effects. Integrating mental health can be an added advantage but might be challenged by limited capacities of the mental health workforce. In this respect a key finding of our study is that mental health intervention was successfully task-shifted to trained HIV-treatment center personnel in a rather remote African setting. We could demonstrate that community health workers involved in counselling and psychosocial supports of PLHIV in the various HIV treatment centers could be trained on appropriate depression screening and management techniques. This model that can effectively expand availability of mental health services for PLHIV has been shown to be feasible and acceptable to nurses, community health workers, and even traditional medicine practitioners in several SSA countries [17, 41, 54]. However, task-shifting mental health intervention in the clinical routine will increase work burden for community health workers who are already overloaded with work in line with other task-shift models of primary health care [53].

This study is limited by the fact that data was collected only from two sites, thus the results cannot be generalized to the whole country. Also, very few participants did viral load test at baseline thus we were unable to ascertain the relationship between treatment adherence and viral suppression. However, this study has provided additional knowledge that supports the importance of depression management for the health and wellbeing of PLHIV and subsequently on their adherence to antiretroviral treatment. Thirdly, the IPT sessions were not done as frequently as recommended but depended on the availability of the participant which could have impacted its administration. However, the intention here was to use non-experts in the domain and not to overburden the participants with more visits out of their treatment drug refill routine. Fourthly, the civil unrest in the English-speaking zones of the country could have affected study participation and/or treatment adherence at SDH as many were internally displaced and those who stayed were faced with movement limitation. However, the adoption of community distribution of antiretroviral drugs and dispensations for multiple months could have minimized this limitation. Further, the questionnaires used in this study (including PHQ9) were administered in both English and French but did not undergo a local tool validation exercise. It is worth noting however that the French version of the questionnaires was reviewed and validated by a habilitated translator in addition to the French version of the PHQ9 questionnaire gotten from a co-investigator of another clinical trial conducted in the Democratic Republic of Congo (a similar context to that of the Cameroonian French speaking population) at that time and whose team had locally validated the questionnaire. Lastly, the minimum calculated sample size of 232 participants with viral load results was not reached despite seeing through 262 participants to 12 months of follow-up and having viral load samples collected from all of them. This potentially affected the power of the analysis in line with the viral suppression outcome.

## Conclusion

The results of this study suggest that routine screening and management of depression as an integral component of HIV care could positively impact HIV treatment outcome. However, there is a need for more research to identify the best combinations of context-specific and cost-effective strategies that can impactfully be integrated in HIV management.

## Supplementary Information

**Additional file 1.** Multi-method adherence measurement tool.

## Data Availability

The datasets used and/or analyzed during the current study are available from the corresponding author on reasonable request.

## Abbreviations

HIV/AIDS: Human Immunodeficiency Virus/Acquired Immune Deficiency Syndrome
PLHIV: People living with HIV
ART: Antiretroviral treatment
PMTCT: Prevention of Mother to Child Transmission
VL: Viral load
CD4: Cluster of differentiation 4
IPT: Interpersonal psychotherapy
SDH: Abong Mbang District Hospital
ADH: Santa District Hospital
R4D: Research for Development International
DZIF: German Center for Infectious Research
SSA: Sub Saharan Africa
WCA: West and Central Africa
UNAIDS: Joint United Nations Programme on HIV/AIDS
