# Supplementary material for: Depression management and antiretroviral treatment outcome among people living with HIV in Northwest and East regions of Cameroon
Source: BMC Infect Dis. 2022 Sep 13;22:732. doi: 10.1186/s12879-022-07711-w (PMC9469586; doi:10.1186/s12879-022-07711-w)
Supplement: Supplementary file 1 — Additional file 1. Multi-method adherence measurement tool. [file 12879_2022_7711_MOESM1_ESM.docx]

**Multi-method Adherence Measurement Tool**

Serial No: ……….… Health Facility: …………….. Date of encounter: ….../.…./…….

Study ID No: …………………………... Visit: M0 M6 M12

Participant: Adult Adolescent (<20yrs) Sex: F M

1. **Drug refill**

1) Number of monthly drug refill appointments respected by participant within the past 6 months? _________ (0-6)

1. **Pill Identification Test (PIT)**

Please kindly inspect the two container and its contents pointing out which of the pills inside is yours. Please also tell me the name of the medication, number of pills to take per dose, the times he or she takes the medication, and whether there are any additional instructions.


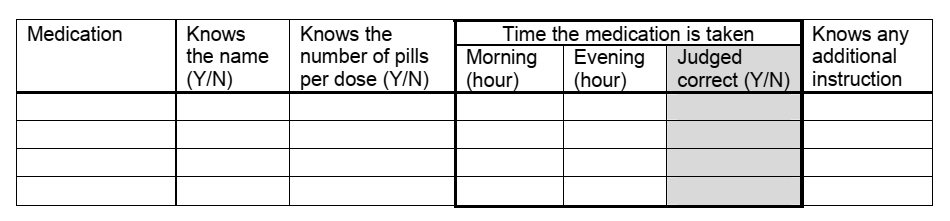


1) In the last 30 days, on how many days did you miss at least one dose of any of your drug

1. **Self-Reporting**

1) Do you sometimes find it difficult to remember to take your medicine? □ No □ Yes

2) When you feel better, do you sometimes stop taking your medicine? □ No □ Yes

3) Thinking back over the past four days, have you missed any of your doses? □ No □ Yes

4) Sometimes do you stop when you feel worse when you take the medicine? □ No □ Yes

1. **Visual Analogue Scale (VAS)**

Please think back with 5 days and identify the times you missed a dose of your drug or took it at the wrong time. By taking into consideration the fact that on the grid below the point 0 (zero) means you missed all your doses and the point 1 (one) means you took all your doses, can you sincerely show me the point between the two that fairly identify your level of adherence?


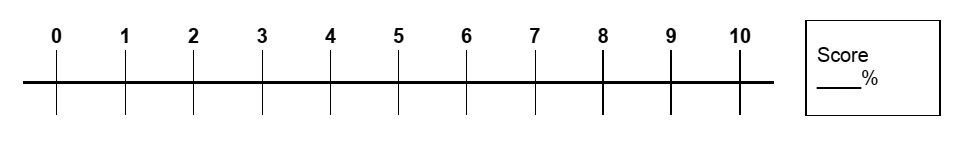


1. **Pill Count**

1) Date of last refill ____________ 2) Expected date of next appointment _______________

3) Actual date of appointment _____________ NB: Drugs finish after 7days of appointment

4) Number of drugs dispensed during last drug refill appointment ________

5) Number of drugs brought back during present drug refill __________

6) Within the number dispensed during drug refill, how many were expected to be taken ____


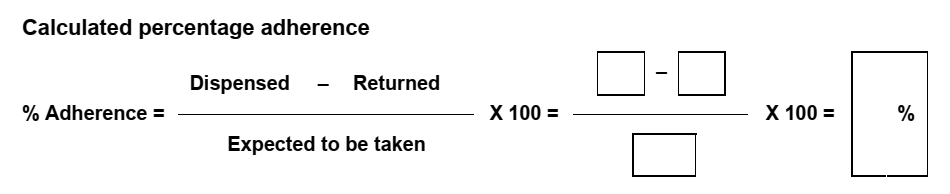


***Assessment guide***

| ***Compliance individual and overall grading*** | | | |
| --- | --- | --- | --- |
| **Drug refill** | Had all 6 refills (3) | Missed 1 refill (2) | Missed 2 or more refills (1) |
| **PIT** | Dose time and instructions (3) | Dose and time only (3) | Dose or time only or confused (1) |
| **Self-reported** | No to all 4 questions (3) | Yes to 1 question (2) | Yes to 2 or more questions (1) |
| **VAS** | 95% and above (3) | 75-94% (2) | Less than 75% (1) |
| **Pill count** | 95% and above (3) | 75-94% (2) | Less than 75% (1) |
| **Overall Compliance** | **Highly compliant (15)** | **Moderately compliant (10-14)** | **Poorly Compliant (5-9)** |

**RESULTS**

Drug Refill score ______ and category ____________

PIT score ____________ and category ____________

Self-reported _________ and category ____________

VAS _______________ and category _____________

Pill count ___________ and category _____________

**Overall adherence score _____________ and category ____________________**
